# Effect of a gel containing pilocarpine on vaginal atrophy in castrated rats

**DOI:** 10.6061/clinics/2016(05)09

**Published:** 2016-05

**Authors:** Cristina A. de Sousa-Lages, Lívio P. de Deus-Lages, Gabriela V. de Sousa, Adinaide C. de Moura-Leal, Airton Mendes Conde, Danylo Rafhael Costa-Silva, Maria da Conceição Barros-Oliveira, Carine Soares Borges, Carla Solange Escórcio-Dourado, Fabiane A. Sampaio, Lívio C. Cunha-Nunes, Benedito B. da-Silva

**Affiliations:** I Universidade Federal do Piauí (UFPI), Escola de Farmácia, Teresina/PI, Brazil; II Universidade Federal do Piauí (UFPI), Departamento de Ginecologia, Teresina/PI, Brazil; III Universidade Federal do Piauí (UFPI), Departamento de Morfologia, Teresina/PI, Brazil; IV Universidade Federal do Piauí (UFPI), Postgraduate Program of the Northeast Network of Biotechnology (RENORBIO), Teresina/PI, Brazil

**Keywords:** Vaginal Atrophy, Pilocarpine, Persistent Estrus, Female Rats, Post-Menopause

## Abstract

**OBJECTIVES:**

To evaluate the effect of Carbopol gel formulations containing pilocarpine on the morphology and morphometry of the vaginal epithelium of castrated rats.

**METHODS:**

Thirty-one female Wistar-Hannover rats were randomly divided into four groups: the control Groups I (n=7, rats in persistent estrus; positive controls) and II (n=7, castrated rats, negative controls) and the experimental Groups, III (n=8) and IV (n=9). Persistent estrus (Group I) was achieved with a subcutaneous injection of testosterone propionate on the second postnatal day. At 90 days postnatal, rats in Groups II, III and IV were castrated and treated vaginally for 14 days with Carbopol gel (vehicle alone) or Carbopol gel containing 5% and 15% pilocarpine, respectively. Next, all of the animals were euthanized and their vaginas were removed for histological evaluation. A non-parametric test with a weighted linear regression model was used for data analysis (*p*<0.05).

**RESULTS:**

The morphological evaluation showed maturation of the vaginal epithelium with keratinization in Group I, whereas signs of vaginal atrophy were present in the rats of the other groups. Morphometric examinations showed mean thickness values of the vaginal epithelium of 195.10±12.23 μm, 30.90±1.14 μm, 28.16±2.98 μm and 29.84±2.30 μm in Groups I, II, III and IV, respectively, with statistically significant differences between Group I and the other three groups (*p*<0.0001) and no differences between Groups II, III and IV (*p*=0.0809).

**CONCLUSION:**

Topical gel formulations containing pilocarpine had no effect on atrophy of the vaginal epithelium in the castrated female rats.

## INTRODUCTION

Menopausal symptoms resulting from ovarian failure, such as hot flashes, bone mass loss, urinary complaints and, principally, vaginal dryness and dyspareunia caused by vaginal atrophy, are common symptoms that cause menopausal women to consult their physicians 1. Estrogen is the most common treatment for vaginal atrophy resulting from hypoestrogenism; however, alternative treatments exist. Recently, CO2 laser therapy has been shown to be a safe and effective option for the treatment of vulvovaginal atrophy symptoms in post-menopausal women 2. This treatment is particularly relevant for women with breast cancer. Other treatments include lubricants for vaginal dryness and dyspareunia 3.

The use of estrogen is contraindicated for the treatment of vaginal atrophy in menopausal women with hormone-dependent cancer; therefore, alternative treatments need to be identified. Pilocarpine chloride is a non-hormonal substance that has attracted the interest of investigators. It is a cholinergic parasympathomimetic agonist with a broad pharmacological effect and predominant muscarinic activity 3,4 that induces an increase in glandular secretion, principally in the salivary and sweat glands 5. Some investigators have demonstrated an improvement in dry mouth and dry eye symptoms as well as an improvement in vaginal dryness in patients with Sjögren’s syndrome who use pilocarpine 6. Nevertheless, other authors have failed to confirm that oral pilocarpine produces an improvement in vaginal dryness humans 7.

The oral use of pilocarpine is associated with undesirable side effects; however, for ethical reasons, studying the application of pilocarpine directly on the vaginal tissue of menopausal women is not possible. Therefore, experimental models need to be identified. From a morphological and endocrine viewpoint, the animal model that is most similar to humans is the female rat 8. The castrated female rat is a biological model with a vaginal epithelium that mimics that of postmenopausal women and therefore, growing interest exists in studying the interaction of various substances in this model. Conversely, the female rat in persistent estrus is a biological model that is characterized by interruption of the estrous cycle and persistent keratinization of the vaginal epithelium due to the effect of constant estrogen production, thus mimicking polycystic ovary syndrome 9,10. Therefore, due to the controversies associated with the relatively few studies that have been conducted and the need for alternatives to estrogen therapy for women with vaginal atrophy and hormone-dependent cancer, the present study was conducted.

## MATERIALS AND METHODS

### Animals

The present study was designed in accordance with the ethical principles established by the Brazilian College of Animal Research and approved by the Animal Research Ethics Committee of the Federal University of Piauí, Brazil (protocol number 012/14). All efforts were made to minimize the number of animals used and to prevent pain, stress or distress in the rats. Thirty-one virgin female Wistar-Hannover rats weighing approximately 200 grams and obtained from the animal laboratory of the School of Veterinary Science, Federal University of Piauí were used in this study. The animals were randomly divided into four groups: Group I: positive controls (n=7 rats in permanent estrus), Group II: negative controls (n=7 castrated rats treated with vehicle only, Carbopol gel), Group III: 5% experimental group (n=8 castrated rats treated with Carbopol gel containing 5% pilocarpine) and Group IV: 15% experimental group (n=9 castrated rats treated with Carbopol gel containing 15% pilocarpine). Permanent estrus was achieved in the animals in Group I by administering a subcutaneous injection of 1.25 mg of testosterone propionate diluted in 0.1 ml of corn oil on the second postnatal day. Estrus was confirmed in the animals at 90 days postnatal based on the total elimination of the external third of the vagina 11 and the presence of keratinization of the vaginal wall epithelium, which are the principal characteristics of persistent estrus that are found during histological examinations at necropsy 9. At 90 days postnatal, the rats in Groups II, III and IV were castrated following a subcutaneous administration of ketamine/midazolam anesthesia. Hypoestrogenism, which is characterized by the absence of mature cells and a predominance of basal cells, was confirmed in Papanicolaou-stained vaginal smears performed 30 days after castration 12. Subsequently, the animals in Group I were given only water and food *ad libitum*, whereas those in Groups II, III and IV also received 0.1 ml of Carbopol gel (the vehicle), Carbopol gel containing 5% pilocarpine or Carbopol gel containing 15% pilocarpine vaginally for 14 days, respectively. On the 15^th^ day, all animals were euthanized. The vaginas were removed through a longitudinal abdominal incision and fixed in 10% buffered formalin for 12-24 hours. The specimens were then dehydrated in increasing ethanol concentrations until reaching absolute ethanol, cleared in xylene, embedded in liquid paraffin and placed in an oven at a temperature of 59°C to produce the tissue blocks 13.

### Morphological and Morphometric Study

For the morphological and morphometric evaluations, 5-μm cross sections were obtained and then the slides were stained with hematoxylin and eosin. An evaluation was conducted using a 400x light microscope connected to a color digital video camera (model SCC-131), which captured and transmitted images to a computer equipped with the Imagelab^®^ software program for image analysis. The morphological evaluation analyzed maturation, cell stratification and the presence of keratin. In the morphometric analysis of the vaginal epithelium, the epithelial thickness was defined as the mean of four linear measurements in µm, measured in the thickest areas 12.

### Statistical analysis

A weighted linear regression model was fitted to compare the mean epithelial thickness of the animals in the four groups. The weight measurements consisted of the inverse of the variance of each group compared to the heteroscedasticity of the control group (*p*<0.05).

## RESULTS

**Figure 1:**
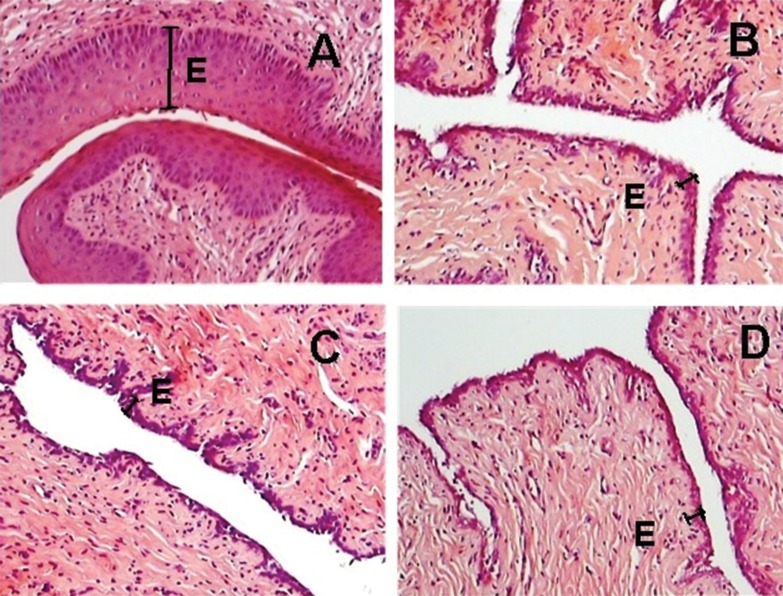
Photomicrographs of histological sections of the vaginal epithelium of rats. Note the thickness of the vaginal epithelium (E) and keratin in rats in persistent estrus (A) and the thin layer of basal and parabasal cells in the castrated rats of Groups II (vehicle) (B), III (5% pilocarpine) (C) and IV (15% pilocarpine) (D). Original magnification 100x.

The morphological analysis of the vaginal epithelium of the rats in Group I (persistent estrus) showed a mature epithelium with a predominance of superficial cells and keratin, whereas in the vaginal epithelium of the castrated animals in Groups II (vehicle), III (5% pilocarpine) and IV (15% pilocarpine) exhibited a predominance of deep cells (basal and parabasal cells) (Figure 1). The morphometric evaluation revealed mean thickness values (±standard deviation) of 195.10±12.23 μm, 30.90±1.14 μm, 28.16±2.98 μm and 29.84±2.30 μm in Groups I, II, III and IV, respectively (Table 1). The mean thickness of the vaginal epithelium of rats in Group I (permanent estrus) was significantly greater than that of animals in Groups II, III or IV (*p*<0.0001), with no statistically significant differences between groups II, III and IV (p=0.0809).

## DISCUSSION

The number of menopausal women is increasing worldwide. In a study conducted in six countries, approximately 45% of menopausal women reported symptoms occurring as a result of ovarian failure 14. Vaginal atrophy, the principal consequence of which is vaginal dryness and dyspareunia, is the most common symptom, affecting between 10 and 50% of menopausal women 15,16. In female breast cancer survivors, the prevalence of vaginal dryness is 61.5%, which is higher than that found in the general population of menopausal women 17. Therefore, we have attempted to identify a non-estrogenic alternative to improve vaginal atrophy and dryness in these women.

These results showed that the thickness of the vaginal epithelium was significantly greater in female rats in permanent estrus than in groups of castrated female rats treated vaginally with Carbopol gel and pilocarpine. In addition, no statistically significant difference was found between the castrated rats that were treated with the vehicle (Carbopol) alone and those that were treated with the different concentrations of pilocarpine.

We are unaware of any other study in which the effect of a Carbopol gel containing pilocarpine was evaluated in the vagina of castrated female rats. Studies on the direct effects of Carbopol gel containing pilocarpine on the urogenital epithelium of women with vaginal atrophy are not possible for ethical reasons. However, in some laboratory animals such as female rats, the anatomical structure and the histology of the urogenital tract are similar to those of humans, rendering these models biologically relevant for the study of factors that are related to female urogenital disorders 18. Unlike the castrated female rats that were used as negative controls in this study due to their ability to mimic menopausal women, female rats in permanent estrus were used as positive controls because the constant estrogenic stimulus is a marked characteristic of polycystic ovary syndrome, and women with this syndrome are affected by a constant influx of estrogen 19.

The most effective therapy used to reduce the symptoms of atrophic vaginitis is estrogen therapy, which is most commonly administered topically, thus avoiding increased exposure to systemic estrogen 20,21. Oral pilocarpine has been associated with toxicity 7; therefore, the vaginal route of administration was selected for the present study. Pilocarpine was incorporated into a Carbopol 940-based gel because it is an inexpensive hydrophilic gel that has the additional advantage of remaining in place for a longer period of time due to its mucoadhesive properties 22,23. The Carbopol gel contained 5% and 15% concentrations of pilocarpine, which are doses that have been used in other studies 6,7,24,25.

Vivino et al. 6 conducted a phase III trial with pilocarpine in patients with Sjögren’s syndrome in which the main endpoint was an improvement in oral and ocular dryness and they also observed a statistically significant decrease in vaginal dryness. Thus, these results suggested that pilocarpine could possibly be used to treat vaginal dryness. The proposed mechanism of action is thought to occur via cholinergic stimulation of Bartholin’s glands, thus increasing mucus production and moisture in the vaginal area 7.

Conversely, Loprinzi et al. 7 conducted a clinical trial and failed to find any beneficial effect of the oral use of pilocarpine for reducing vaginal dryness in women with vaginal atrophy. The results of these investigators are consistent with the findings of the present study, which showed no detectable effect of vaginal pilocarpine on atrophy of the vaginal epithelium of castrated female rats. Further studies are needed to identify other alternative treatments.

## AUTHOR CONTRIBUTIONS

De Sousa Lages CA, de Deus-Lages LP, de Sousa GV, de Moura-Leal AC and Conde Junior AM conceived and designed the study and wrote the final version of the manuscript. Costa-Silva DR, Barros-Oliveira MC, Borges CS, Escorcio-Dourado CS, Sampaio FA, Cunha-Nunes LC, and da Silva BB were responsible for conducting the study, statistically analyzing the data and drafting and revising the manuscript.

## Figures and Tables

**Table 1:** Means and standard deviations of the vaginal epithelium thickness of female rats in persistent estrus (Group I, positive control) and the castrated rats in Groups II (negative control), III and IV (experimental groups) treated with gels with vehicle and pilocarpine.

		Epithelial thickness (µm)	
Group	n	Mean	SD
I	7	195.10*	12.23
II	7	30.90	1.14
III	8	28.16	2.98
IV	9	29.84	2.30

Group I exhibited a significantly greater epithelial thickness than Groups II, III and IV (*p*<0.0001) and no difference was observed between Groups II, III and IV (*p*=0.0809).
